# Separation effectiveness of ideal ion exchange membranes: Application of the Gibbs-Donnan theory

**DOI:** 10.1371/journal.pone.0317818

**Published:** 2025-01-28

**Authors:** Jacek Waniewski, Leszek Pstras, Mauro Pietribiasi

**Affiliations:** Nalecz Institute of Biocybernetics and Biomedical Engineering, Polish Academy of Sciences, Warsaw, Poland; Jeddah University: University of Jeddah, SAUDI ARABIA

## Abstract

Ion exchange membranes (IEMs) are permselective membranes that, in principle, only allow the flow of ions with a specific charge sign, opposite to that of the fixed membrane ionic groups (counter-ions). This charge-based selectivity, like the size-based selectivity of classic semipermeable membranes, leads to an uneven distribution of permeating ions on the two sides of the membrane, which allows for ion separation or recovery in various processes in industry or environmental protection. Here, we apply the principles of mass balance, charge neutrality, and equality of electrochemical potentials in the state of thermodynamic equilibrium to provide a simple method for estimating the Gibbs-Donnan factors and the equilibrium concentrations of permeating ions in two compartments separated by an ideal IEM, i.e. an IEM that is not permeable to co-ions. We present the method for the case when the equilibrium concentrations are known in one compartment and need to be estimated in the other compartment as well as for the case when the total masses of ions in both compartments are known and their equilibrium concentrations need to be predicted. For both cases, the presented nonlinear algebraic equations require in general the use of numerical methods to approximate their mathematical solutions, although we present as well some closed solutions for simple cases with ideal ionic mixtures. Based on the extended Debye–Hückel theory, we also provide analogous equations (general and for specific cases) for systems with non-ideal ionic mixtures. The presented method can provide the expected ideal separation effectiveness of an IEM, which can then be used to assess the relative separation effectiveness of a real membrane.

## 1. Introduction

The phenomenon of Gibbs-Donnan equilibrium in two ionic solutions separated by a permselective membrane consists in the inequality of activities (or concentrations for ideal solutions) of permeating ions on the two sides of the membrane, when some non-permeating charged species are present in one or both solutions with different total charge on the two sides of the membrane [1,2]. Frequently, the selectivity of a membrane (i.e. its permeability to some species only) is based on the size of its pores relative to the size of ions or molecules present in the solutions, as is the case, for example, with physiological membranes [3,4]. However, membrane selectivity can also be based on the charge of the ionic functional groups within the membrane that attract oppositely-charged ions (counter-ions) and repel similarly-charged ions (co-ions), as in ion exchange membranes (IEM). Therefore, the Gibbs-Donnan equilibrium principle does also apply to IEMs or ion exchange resins [5,6].

Experimental studies on the function of IEMs are typically performed by placing some ions of interest in two fluid compartments separated by the studied membrane and then sampling both fluids to monitor changes in the concentrations (or activities) of those ions on the two sides of the membrane [5,7–10]. Based on such an experiment and using a thermodynamic kinetic model of transmembrane ion transport, the corresponding transport coefficients of the membrane (e.g. the diffusion coefficient or permselectivity index) can be estimated by fitting the model to experimental data [5,8,10–13] (similarly as it can be done for non-charged permselective membranes, e.g. for the human peritoneal membrane during peritoneal dialysis [14]).

The approach we present in this study can provide useful *a priori* information on such experiments. In particular, we present and discuss a relatively simple method for estimating the expected (ideal) equilibrium concentrations of permeating ions on the two sides of an IEM based on the initial concentrations of all ions and the compartment volumes. Using this method, one may estimate the (ideal) equilibrium concentrations without having to wait until the experimental system reaches that equilibrium, which may be particularly useful in time-constrained frameworks. Moreover, such *a priori* information on the ideal separation of permeating ions (to be obtained after a sufficiently long time) and the equilibrium Gibbs-Donnan factors may be helpful in the design of experiments and industrial processes involving IEMs. The wide range of applications of IEMs, in particular for the so-called Donnan dialysis, which is extensively used for separation, removal, or recovery of various substances in industry, environment protection, food processing, desalination and water treatment, among others [15–18], further supports the need for a simple method for evaluating different configurations of driving and feed ions for such systems to establish the configuration that can provide the maximal efficiency of ion exchange during Donnan dialysis.

To this end, here we present an extension of our previous work on the calculation of the Gibbs-Donnan factors for multi-ion solutions separated by a permselective membrane [19] but for the case where the membrane selectivity is due to fixed charged groups within the membrane, as opposed to classic size-based selectivity. Our considerations are valid only for ideal IEMs, i.e. membranes permeable only to ions with a charge opposite to the fixed charge of the membrane, i.e. counter-ions (here, we do not consider ion-specific IEMs, i.e. we only consider IEMs that allow the passage of any counter-ions, thus not discriminating between like-charged ions with different charge numbers or between different ions with the same charge number). In reality, IEMs are never perfect and are always somewhat permeable also to generally non-permeating ions (co-ions). Systems with such non-ideal IEMs may transiently reach a state close to the ideal equilibrium, but in the long term they tend to an equilibrium resulting from partial leakage of co-ions that were not expected to pass through the membrane. For such real IEMs, our considerations characterize, therefore, not the membrane per se but the distribution of ions on the two sides of a similar but ideal membrane, and hence provide the theoretical maximal ion separation effectiveness. Then, the relative separation effectiveness of a real membrane may be defined as the ratio of ion separation achieved in reality (e.g. in an experimental study) over the maximal possible separation for an ideal IEM.

## 2. Theoretical derivations

### 2.1. Gibbs-Donnan equilibrium for IEM

Let us consider two compartments separated by an ideal IEM that allows the passage of ions with a specific charge sign (permeating ions, counter-ions) and is impermeable to ions with the opposite charge sign (non-permeating ions, co-ions). The system is assumed to be isothermal.

The solution in each compartment contains n species of permeating ions with the same charge sign (counter-ions to the fixed charge groups within the membrane), each with a charge number (ion valency) z_i_ and molar concentration c_i_, i = 1,2,…n, as well as m species of non-permeating (np) ions with the opposite charge sign (co-ions to the fixed charge groups within the membrane), each with a charge number z_β_ and molar concentration c_β_ (fixed and known), β = 1,2,…m.

The non-permeating ions may be collectively described by the product $Z_{np}C_{np}=\sum_{\beta =1}^{m}z_{\beta }c_{\beta }$, where Z_np_ is their average (molar concentration-weighted) charge number and C_np_ is their total molar concentration (fixed in each compartment).

The electroneutrality of the solution in each compartment requires:

$$\sum_{i=1}^{n}z_{i}c_{i,1}+Z_{np,1}C_{np,1}=0$$ (1)

$$\sum_{i=1}^{n}z_{i}c_{i,2}+Z_{np,2}C_{np,2}=0$$ (2)

for compartments 1 and 2, respectively.

Equilibrium of two multi-ion solutions separated by a permselective membrane requires the intercompartmental equilibration of the electrochemical potentials of each permeating ion [2,4,19].

At equilibrium, the ratio of activities of a permeating ion i (a_i_) in two fluid compartments separated by a permselective membrane is related to the Nernst potential as follows [2,4,19]:

$$V^{Nernst}=-\frac{RT}{z_{i}F}\ln\left(\frac{a_{i,2}}{a_{i,1}}\right)$$ (3)

where R is the perfect gas constant, T–the absolute temperature of the mixture, and F–the Faraday constant.

Therefore, for each permeating ion i, the ratio of its activities in compartments 1 and 2 can be expressed as follows:

$${\left(\frac{a_{i,2}}{a_{i,1}}\right)}^{1/z_{i}}=\exp\left(-\frac{FV^{Nernst}}{RT}\right)=const$$ (4)

and has the same value for all i = 1, 2,…, n.

In all further considerations we assume non-ideal solutions in which ion activity is proportional to ion concentration a_i_ = k_i_c_i_, with the activity coefficient k_i_ that depends on the thermodynamic state of the system, in particular on the concentrations of all ions in the mixture. We assume here that all values of k_i_ are known as functions of the composition of the solution (from theoretical or empirical investigations). The presented theory is also directly applicable to ideal solutions with all k_i_ = 1.

Based on the above assumptions and Eq (4), for any two charge numbers z_i_ and z_j_ (i, j = 1,2, …n), we have:

$${\left(\frac{k_{i,2}c_{i,2}}{k_{i,1}c_{i,1}}\right)}^{1/z_{i}}={\left(\frac{k_{j,2}c_{j,2}}{k_{j,1}c_{j,1}}\right)}^{1/z_{j}}$$ (5)

Let us now assume that the equilibrium concentrations of all permeating ions in compartment 1 are known and that we want to calculate the equilibrium concentrations in compartment 2. Let us also select the permeating ions with the charge number z_1_ as a reference ion species (note that this selection is entirely arbitrary and that other permeating ions present in the mixture could also be selected as a reference species). Then, using Eq (5):

$$c_{i,2}=K_{1}^{i}{\left(\frac{c_{1,2}}{c_{1,1}}\right)}^{z_{i}/z_{1}}c_{i,1}$$ (6)

for all i = 1,2, …,n, where $K_{1}^{i}={\left(\frac{k_{1,2}}{k_{1,1}}\right)}^{z_{i}/z_{1}}\frac{k_{i,1}}{k_{i,2}}$. Note that $K_{1}^{1}=1$.

Therefore, the problem can be reduced to calculation of the ratio x = c_1,2_/c_1,1_ or, equivalently, calculation of c_1,2_ (since c_1,1_ is known). Using Eqs (2) and (6) we have:

$$\sum_{i=1}^{n}x^{z_{i}/z_{1}}K_{1}^{i}\gamma_{i}+\gamma_{np,2}=0$$ (7)

where γ_i_ = z_i_c_i,1_/(z_1_c_1,1_) and γ_np,2_ = Z_np,2_ C_np,2_/(z_1_ c_1,1_) are ionic equivalents expressed relative to the equivalent of the selected reference ion in compartment 1. Note that γ_1_ = 1.

Note also that if all ions could pass through the membrane, i.e., if γ_np,1_ = γ_np,2_ = 0 (for a hypothetical completely ineffective IEM), Eq (7) would have the mathematical solution x = 1 (with all $K_{1}^{i}=1$), because for these conditions Eq (7) would be reduced to Eq (1). This mathematical solution x = 1 means equilibration of the concentrations of all ions between compartments 1 and 2.

To avoid negative exponents in Eq (7), let us introduce z_max_ being the maximal value of z_i_ and -z_i_. Then:

$$\sum_{i=1}^{n}x^{\left(z_{\max}+z_{i}\right)/z_{1}}K_{1}^{i}\gamma_{i}+\gamma_{np,2}x^{z_{\max}/z_{1}}=0$$ (8)

is the polynomial equation that can be solved in closed formulas for some simple and ideal mixtures of ions (with all $K_{1}^{i}$ equal to 1). For example, if n = 1 we get x = -γ_np,2_, because $K_{1}^{1}=1$ and γ_1_ = 1; note that here x is independent of the activity coefficients.

Furthermore, if n (the number of distinct permeating ions) is higher than 1, from Eq (1) we get:

$$\gamma_{n}=-\left(\sum_{i=1}^{n-1}\gamma_{i}+\gamma_{np,1}\right)$$ (9)

where *γ*_n_ = z_n_c_n,1_/(z_1_c_1,1_)_,_ *γ*_i_ = z_i_c_i,1_/(z_1_c_1,1_), and *γ*_np,1_ = Z_np,1_C_np,1_/(z_1_c_1,1_), and then:

$$\sum_{i=1}^{n-1}K_{1}^{i}\gamma_{i}x^{\left(z_{\max}+z_{i}\right)/z_{1}}-K_{1}^{n}\left(\sum_{i=1}^{n-1}\gamma_{i}+\gamma_{np,1}\right)x^{\left(z_{\max}+z_{n}\right)/z_{1}}+\gamma_{np,2}x^{z_{\max}/z_{1}}=0$$ (10)

Closed solutions for such nonlinear algebraic equations as Eq (10) are in general not known (note that the exponents of x may be rational numbers, not necessarily integers, and even for integer exponents of x, general closed solutions are known only up to the third order, i.e. for cubic equations; furthermore, the number of ions is arbitrary) and they are typically solved by numerical methods for each case separately. All equations in this study are solved using the trust-region-dogleg algorithm implemented in the routine *fsolve* with functional tolerance 10^−12^ and step tolerance 10^−12^ in Matlab R2021a. There are however special simple cases of closed mathematical solutions and some of them are presented in this paper. An example of the numerical solutions of Eq (10) is shown in Section 5, Example 1.

The Gibbs-Donnan factor (often called simply Donnan factor) for ions with the charge number z_i_ is defined as:

$$DF_{i,21}=c_{i,2}/c_{i,1}$$ (11)

and can be calculated as follows, if Eq (10) is solved for x:

$$DF_{i,21}=K_{1}^{i}x^{z_{i}/z_{1}}$$ (12)

The definition (11) allows for calculation of c_i,2_ if DF_i,21_ and c_i,1_ are known:

$$c_{i,2}=DF_{i,21}c_{i,1}$$ (13)

In the above, we assume that the IEM has a much higher charge density compared to the bulk charge of either of the solutions and that there is no diffusion potential between the two sides of the membrane (otherwise, the diffusion potential should be accounted for, and the Gibbs-Donnan equilibrium should be considered separately for the two interfaces between the solution and the membrane, as in the Teorell-Meyer-Sievers theory [20]).

We also assume that none of the considered permeating ions is adsorbed on the membrane, which not only could directly alter the Gibbs-Donnan equilibrium but also could alter the charge-exclusion properties of the membrane.

Note that in the general case all $K_{1}^{i}$ may depend on x (except for $K_{1}^{1}=1$), and hence solving Eq (10) is not trivial and usually requires a numerical solution, especially for multi-ion mixtures or when the exponents of x are not integers but rational numbers (even for integer exponents of x, closed solutions may be known only up to the third order, i.e. for cubic equations). An example of such numerical solutions of Eq (10) is shown in Section 5, Example 1. There are, however, some special simple cases with closed mathematical solutions of this equation, some of which are presented in the section 2.3.

### 2.2. Effectiveness factor

The effectiveness factor (EF) for the separation of a permeating ion i may be defined in general as the difference in ion concentration in the (receiver) compartment 1 and the (feed) compartment 2 divided by its concentration in the receiver compartment 1:

$$EF_{i}=\frac{c_{i,1}-c_{i,2}}{c_{i,1}}$$ (14)

For an ideal IEM and the state of equilibrium in the system, using the definition of the Gibbs–Donnan factor, Eq (11), one obtains:

$$EF_{i}=1-DF_{i,21}$$ (15)

For experimental studies, the effectiveness of ion separation or recovery using an IEM is often described by the so-called kinetic efficiency factor (kEF) that relates the concentration of feed ions in the feed compartment at time t (c_feed,t_) to their initial concentration in the feed compartment (c_feed,0_), $kEF\left(t\right)=1-c_{feed,t}/c_{feed,0}$[21]. If we denote the value of kEF when the system reached the equilibrium as kEF_eq_ and the ratio of volumes of the receiver, V_rec_, and feed, V_feed_, compartments as b, b = V_rec_/V_feed_, then $kEF_{eq}=b/(1+b-EF)$ for the case of the feed ions being initially present only in the feed compartment, where EF is given by Eq (14) (for feed ions). The maximal attainable value of kEF corresponds to the Gibbs–Donnan equilibrium for the given configuration of feed and driving ions.

### 2.3. Special cases

#### Case 1

Let us assume that all permeating ions have the same charge number z. Then, Eq (10) takes a simpler form:

$$x\left(\sum_{i=1}^{n-1}K_{1}^{i}\gamma_{i}-K_{1}^{n}\left(\sum_{i=1}^{n-1}\gamma_{i}+\gamma_{np,1}\right)\right)+\gamma_{np,2}=0$$ (16)

and therefore:

$$x=-\gamma_{np,2}/\left(\sum_{i=1}^{n-1}K_{1}^{i}\gamma_{i}-K_{1}^{n}\left(\sum_{i=1}^{n-1}\gamma_{i}+\gamma_{np,1}\right)\right)$$ (17)

If the coefficients K depend on x, then the above equation must be solved numerically. However, for ideal solutions with all $K_{1}^{i}$ equal to 1, Eq (17) yields:

$$x=\gamma_{np,2}/\gamma_{np,1}$$ (18)

#### Case 2

Let us consider now permeating ions of two different charge numbers: 1 and 2 or -1 and -2 (both positive or both negative). Then, for abs(z_1_) = 1, Eq (10) becomes:

$$x-K_{1}^{2}\left(1+\gamma_{np,1}\right)x^{2}+\gamma_{np,2}=0$$ (19)

and after rearrangement:

$$K_{1}^{2}\left(1+\gamma_{np,1}\right)x^{2}-x-\gamma_{np,2}=0$$ (20)

If one assumes that $K_{1}^{2}$ is independent of x then the mathematical solution is:

$$x=\frac{1-\sqrt{1+4\gamma_{np,2}K_{1}^{2}\left(1+\gamma_{np,1}\right)}}{2K_{1}^{2}\left(1+\gamma_{np,1}\right)}$$ (21)

The Gibbs–Donnan factors are DF_1,21_ = x and $DF_{2,21}=K_{1}^{2}x^{z_{2}/z_{1}}$. Note that *γ*_np,2_ < 0 and *γ*_np,1_ < -1.

## 3. Non-ideal solutions: Activity coefficients dependent on ionic strength

A frequently used and studied description of ionic activity coefficients is based on the theory proposed by Debye and Hückel in 1923 [22] with further theoretical and phenomenological extensions by other authors [23,24]. In general, this theory is based on the assumption that activity coefficients k_i_ depend mainly on the ion charge number z_i_, the effective ion radius r_i_, and ionic strength of the solution defined as $I=\frac{1}{2}\sum z_{i}^{2}c_{i}$ with the summation over all ions present in the solution (k_i_ depend also on the type of solvent, temperature, and pressure) [23,24].

For our system, the ionic strengths in compartments 1 and 2 are:

$$I_{1}=\frac{1}{2}\sum_{i=1}^{n}z_{i}^{2}c_{i,1}+\frac{1}{2}\sum_{\beta =1}^{m}z_{\beta }^{2}c_{\beta ,1}$$ (22)

$$I_{2}=\frac{1}{2}\sum_{i=1}^{n}z_{i}^{2}c_{i,2}+\frac{1}{2}\sum_{\beta =1}^{m}z_{\beta }^{2}c_{\beta ,2}$$ (23)

Note that non-permeating ions may be different in the two considered compartments and that their concentrations in each compartment are fixed and known. According to our assumption, the concentrations of permeating ions in compartment 1 are also fixed and known, and therefore l_1_ can be directly calculated. However, for l_2_ one needs to apply Eq (6), which gives:

$$I_{2}=\frac{1}{2}\sum_{i=1}^{n}z_{i}^{2}K_{1}^{i}\left(I_{2}\right)x^{z_{i}/z_{1}}c_{i,1}+\frac{1}{2}\sum_{\beta =1}^{m}z_{\beta }^{2}c_{\beta ,2}$$ (24)

We assume here that the ionic strength of the solution is the only factor influencing the activity coefficients k_i_(the other factors being fixed and known). Therefore, the coefficients $K_{1}^{i}\left(I_{2}\right)$ are known functions of one unknown l_2_. Thus, if we denote this unknown by w and use Eqs (7) and (24), then:

$$\sum_{i=1}^{n}K_{1}^{i}\left(w\right)x^{z_{i}/z_{1}}\gamma_{i}+\sum_{\beta =1}^{m}\gamma_{\beta ,2}=0$$ (25)

$$\overset{\text{n}}{\underset{\text{i}=1}{\sum }}{\text{z}}_{i}{\text{K}}_{1}^{\text{i}}\left(\text{w}\right){\text{x}}^{{\text{z}}_{\text{i}}/{\text{z}}_{1}}\gamma_{\text{i}}+\overset{\text{m}}{\underset{\beta =1}{\sum }}{\text{z}}_{\beta }\gamma_{\beta ,2}-\frac{2\text{w}}{{\text{z}}_{1}{\text{c}}_{1,1}}=0$$ (26)

are two equations with two unknowns x and w, where γ_β,2_ = z_β_ c_β,2_/(z_1_ c_1,1_). Note that if $K_{1}^{i}\left(w\right)=1$ for all i = 1, …, n, then w can be calculated from Eq (26), if x is found using Eq (25).

For permeating ions with the same charge number (z_i_ = z_1_ for all i), Eq (25) multiplied by z_1_ gives:

$${\text{z}}_{1}\overset{\text{n}}{\underset{\text{i}=1}{\sum }}{\text{K}}_{1}^{\text{i}}\left(\text{w}\right){\text{x}}^{{\text{z}}_{\text{i}}/{\text{z}}_{1}}\gamma_{\text{i}}+{\text{z}}_{1}\overset{\text{m}}{\underset{\beta =1}{\sum }}\gamma_{\beta ,2}=0$$ (27)

and from Eq (26) we have:

$${\text{z}}_{1}\overset{\text{n}}{\underset{\text{i}=1}{\sum }}{\text{K}}_{1}^{\text{i}}\left(\text{w}\right){\text{x}}^{{\text{z}}_{\text{i}}/{\text{z}}_{1}}\gamma_{\text{i}}+\overset{\text{m}}{\underset{\beta =1}{\sum }}{\text{z}}_{\beta }\gamma_{\beta ,2}-\frac{2\text{w}}{{\text{z}}_{1}{\text{c}}_{1,1}}=0$$ (28)

Subtracting Eq (27) from Eq (28) gives:

$$\overset{\text{m}}{\underset{\beta =1}{\sum }}{\text{z}}_{\beta }\gamma_{\beta ,2}-\frac{2\text{w}}{{\text{z}}_{1}{\text{c}}_{1,1}}-{\text{z}}_{1}\overset{\text{m}}{\underset{\beta =1}{\sum }}\gamma_{\beta ,2}=0$$ (29)

or:

$$\text{w}=\left(\sum_{\beta =1}^{\text{m}}{\text{z}}_{\beta }^{2}{\text{c}}_{\beta ,2}-{\text{z}}_{1}\sum_{\beta =1}^{\text{m}}{\text{z}}_{\beta }{\text{c}}_{\beta ,2}\right)/2$$ (30)

Note that if all permeating ions have the same charge number, then it follows from Eq (30) that l_2_ = w is independent of x.

If we additionally assume that all co-ions have the same charge number, then $w=\left(z_{\beta }^{2}-z_{1}z_{\beta }\right)\overset{m}{\underset{\beta =1}{\sum }}c_{\beta ,2}/2$. In particular, if all z_β_ = -z_1_, then $\text{w}={\text{z}}_{1}^{2}\overset{\text{m}}{\underset{\beta =1}{\sum }}{\text{c}}_{\beta ,2}$, or, if all z_β_ = -2z_1_ then $\text{w}=3{\text{z}}_{1}^{2}\overset{\text{m}}{\underset{\beta =1}{\sum }}{\text{c}}_{\beta ,2}$, and in general, if z_β_ = -nz_1_ then $\text{w}=\frac{1}{2}\text{n}\left(\text{n}+1\right){\text{z}}_{1}^{2}\overset{\text{m}}{\underset{\beta =1}{\sum }}{\text{c}}_{\beta ,2}$. Furthermore, if all z_β_ = -z_1/n_, then $\text{w}=\frac{1}{2\text{n}}\left(1+\frac{1}{\text{n}}\right){\text{z}}_{1}^{2}\overset{\text{m}}{\underset{\beta =1}{\sum }}{\text{c}}_{\beta ,2}$; for example, if z_1_ = 2 and all z_β_ = -1, then $w=\frac{3}{2}\sum_{\beta =1}^{m}c_{\beta ,2}$.

For a more general case, where the activity coefficients k_i_ depend not only on ionic strength, we can estimate them using the extended Debye–Hückel theory [23,24]:

$$\ln\left(k_{i}\left(I\right)\right)=\frac{Az_{i}^{2}\sqrt{I}}{1+Br_{i}\sqrt{I}}$$ (31)

where parameters A and B depend on solvent permittivity, temperature, and pressure, and r_i_ is the minimal distance of approach to ion i (meaning that other ions cannot get any closer) [23,24]. Assuming that the products Br_i_ are the same for all ions (Br_i_ = D) [23,24], from the definition of $K_{1}^{i}$, see Eq (6), we get:

$$K_{1}^{i}=\exp\left(z_{i}\left(z_{i}-z_{1}\right)\frac{A\left(\sqrt{I_{2}}-\sqrt{I_{1}}\right)}{\left(1+D\sqrt{I_{2}}\right)\left(1+D\sqrt{I_{1}}\right)}\right)$$ (32)

The assumption of Br_i_ = D is used here, following other researchers [23,24], for simplicity, to enable easy application of the method for different ions. If the minimal distances of approach to individual ions (r_i_) differ substantially, the true value of parameter $K_{1}^{i}$ may differ from that calculated using Eq (32). If this theory were to be applied for any particular system, the values r_i_ of all involved ions should be obtained from the literature or estimated from new experimental data.

Then we can solve Eqs (25), (26) and (32) for x and l_2_ = w. For example, in Table 1 we show a few exemplary numerical solutions of such a system for the case of an ideal cation exchange membrane with two types of permeating cations (counter-ions): monovalent (e.g. Na^+^) and bivalent (e.g. Ca^++^) and one monovalent non-permeating co-ion (e.g. Cl^–^).

**Table 1:** Examples of the Gibbs-Donnan factors for non-ideal solutions separated by an ideal cation exchange membrane. The Table presents the Gibbs–Donnan factors (DF_1,21_ and DF_2,21_) and the coefficients $K_{1}^{2}$ describing the equilibrium across an ideal cation exchange membrane obtained by solving Eqs (25), (26) and (32) for l_2_ = w for three different sets of concentrations of permeating ions (counter-ions) in compartment 1 and non-permeating ions (co-ions) in both compartments. The assumed values of A and D were 1.172 (L/mol)^1/2^ and 1.5 (L/mol)^1/2^, respectively [23]. The considered system includes two types of permeating cations: monovalent (z_1_ = 1) with concentration c_1,1_ in compartment 1 and bivalent (z_2_ = 2) with concentration c_2,1_ in compartment 1, and one type of monovalent non-permeating anion (Z_np_ = -1) with concentration C_np,1_ in compartment 1 and C_np,2_ in compartment 2.

Known equilibrium concentrations, mol/L				Parameters describing the Gibbs-Donnan equilibrium		
c_1,1_	c_2,1_	C_np,1_	C_np,2_	$K_{1}^{2}$	DF_1,21_	DF_2,21_
0.01	0.02	0.05	0.01	0.805	0.423	0.144
0.1	0.2	0.5	0.1	0.723	0.440	0.140
1	2	5	1	0.768	0.431	0.142

Note that in the examples presented in Table 1 the pairwise ratios between the four assumed concentrations are the same in all three considered cases. This means that the coefficients *γ* (i.e. *γ*_i_,*γ*_np,1_, and *γ*_np,2_) are the same in all those three cases, and hence, if we considered ideal solutions ($K_{1}^{2}=1$), the Gibbs-Donnan factors would be the same in all those cases: DF_1,21_ = 0.390 and DF_2,21_ = 0.152.

## 4. Distribution of permeating ions between two compartments at equilibrium

In many processes involving IEMs, one knows the total mass of each permeating ion species (counter-ions) in the whole system and the total mass or equivalents of non-permeating ions (co-ions) in each compartment. An electroneutral system with an ideal IEM would tend to the equilibrium described by Eq (5), with the balance of charge described by Eqs (1) and (2), and the balance of mass for each ion as follows:

$$c_{i,1}V_{1}+c_{i,2}V_{2}=M_{i}$$ (33)

where V_j_, j = 1, 2, denotes the equilibrium volume of compartment j and M_i_ denotes the total mass of ion i in the system.

Let us assume that V_1_, V_2_ and M_i_ for all i are known and that the equilibrium concentrations in the two compartments, c_i,1_ and c_i,2_, need to be found for all permeating ions.

Using the Gibbs–Donnan factor one gets from Eq (33):

$$c_{i,1}V_{1}+DF_{i,21}c_{i,1}V_{2}=M_{i}$$ (34)

and therefore:

$$c_{i,1}=\frac{M_{i}}{V_{1}+DF_{i,21}V_{2}}$$ (35)

Furthermore, from Eq (33):

$$c_{i,2}=\frac{M_{i}-c_{i,1}V_{1}}{V_{2}}$$ (36)

For ideal solutions in which all permeating ions have the same charge number (all z_i_ = z), the above problem can be easily solved, given that in such a case DF_i,21_ depend only on the ratio of equivalents of non-permeating ions on the two sides of the membrane, see Eq (18). Therefore:

$$c_{i,1}=\frac{Z_{np,1}C_{np,1}V_{1}}{Z_{np,1}C_{np,1}V_{1}+Z_{np,2}C_{np,2}V_{2}}\frac{M_{i}}{V_{1}}$$ (37)

However, in the general case (with permeating ions with different charge numbers), the Gibbs–Donnan factors DF_i,21_ depend also on the equivalents of all permeating ions. In such cases, all equations for charge and mass balance (i.e. Eqs (1), (2) and (34)) need to be solved together.

For non-ideal solutions, using Eq (12), one gets from Eq (35):

$$c_{i,1}=\frac{h_{i}}{b+K_{1}^{i}x^{z_{i}/z_{1}}}$$ (38)

where h_i_ = M_i_/V_2_ is the hypothetical concentration of ions i if they were present in compartment 2 only (or the true, initial concentration, if ions i were initially present in compartment 2 only), and b = V_1_/V_2_. Therefore, coefficients γ from Eq (7) may be expressed as follows:

$$\gamma_{i}=\frac{z_{i}h_{i}}{z_{1}h_{1}}\frac{b+x}{b+K_{1}^{i}x^{z_{i}/z_{1}}}$$ (39)

and:

$$\gamma_{np,2}=\frac{Z_{np,2}C_{np,2}}{z_{1}h_{1}}\left(b+x\right)$$ (40)

Finally, Eq (7) may be written as:

$$\left(b+x\right)\left(\sum_{i=1}^{n}K_{1}^{i}\frac{z_{i}h_{i}}{z_{1}h_{1}}\frac{x^{z_{i}/z_{1}}}{b+K_{1}^{i}x^{z_{i}/z_{1}}}+\frac{Z_{np,2}C_{np,2}}{z_{1}h_{1}}\right)=0$$ (41)

or equivalently:

$$\sum_{i=1}^{n}K_{1}^{i}\phi_{i}\frac{x^{z_{i}/z_{1}}}{b+K_{1}^{i}x^{z_{i}/z_{1}}}+\phi_{np,2}=0$$ (42)

where $\phi_{i}=z_{i}h_{i}/\left(z_{1}h_{1}\right)=z_{i}M_{i}/\left(z_{1}M_{1}\right)$ and $\phi_{np,2}=Z_{np,2}C_{np,2}/\left(z_{1}h_{1}\right)$.

For ideal solutions, if all permeating ions have the same charge number, Eq (42) may be solved as:,

$$x=-b\frac{\phi_{np,2}}{\sum_{i=1}^{n}\phi_{i}+\phi_{np,2}}$$ (43)

When n = 1, the Eq (42) has the mathematical solution $x=-b\frac{\phi_{np,2}}{1+\phi_{np,2}}$, which is valid for both ideal and non-ideal solutions.

Another frequently studied case is a cation exchange membrane with a mixture of monovalent cations (z_1_ = 1) and other cations with the same charge number z_2_ > 1 (n = 2) [13,25]. In this case, the general Eq (42) can be reduced to:

$$\phi_{1}K_{1}^{1}\frac{x}{b+K_{1}^{1}x}+\phi_{2}K_{1}^{2}\frac{x^{z_{2}}}{b+K_{1}^{2}x^{z_{2}}}+\phi_{np,2}=0$$ (44)

Note that ϕ_1_ = 1 and $K_{1}^{1}=1$ (by definition), and hence Eq (44) can be further reduced to:

$$K_{1}^{2}\left(1+\phi_{2}+\phi_{np,2}\right)x^{z_{2}+1}+K_{1}^{2}b\left(\phi_{2}+\phi_{np,2}\right)x^{z_{2}}+b\left(1+\phi_{np,2}\right)x+b^{2}\phi_{np,2}=0$$ (45)

which in general needs to be solved numerically. An example of numerical solutions of Eq (45) is presented in Section 5, Example 1.

If we use the general description of $K_{1}^{i}=K_{1}^{i}\left(w\right)$ based on the Debye–Hückel theory with only one unknown parameter w = I_2_, see Eq (32), then we need to solve two equations for two variables x and w, i.e. Eqs (26) and (42):

$$\sum_{i=1}^{n}K_{1}^{i}\left(w\right)\phi_{i}\frac{x^{z_{i}/z_{1}}}{b+K_{1}^{i}\left(w\right)x^{z_{i}/z_{1}}}+\phi_{np,2}=0$$ (46)

$$\overset{\text{n}}{\underset{\text{i}=1}{\sum }}{\text{z}}_{\text{i}}{\text{K}}_{1}^{\text{i}}\left(\text{w}\right){\text{x}}^{{\text{z}}_{\text{i}}/{\text{z}}_{1}}\gamma_{\text{i}}+\overset{\text{m}}{\underset{\beta =1}{\sum }}{\text{z}}_{\beta }\gamma_{\beta ,2}-\frac{2\text{w}}{{\text{z}}_{1}{\text{c}}_{1,1}}=0$$ (47)

## 5. Examples

**Example 1.** *Permeating cations with two different charge numbers (a theoretical case)*

**Fig 1:**
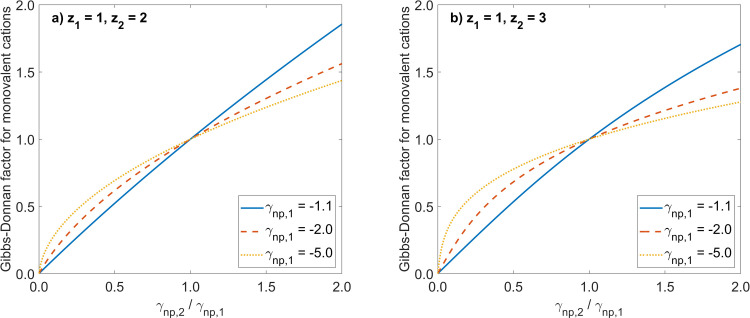
Gibbs-Donnan factors for different ratios of non-permeating ions on the two sides of a cation exchange membrane. Shown are the relationships between the Gibbs-Donnan factor for a permeating monovalent cation and the ratio of the equivalents of non-permeating ions on the two sides of a cation exchange membrane, $\gamma_{\text{np},\text{2}}/\gamma_{\text{np},\text{1}}=Z_{np,2}C_{np,2}/Z_{np,1}C_{np,1}$, calculated according to Eq (10) for different values of *γ*_np,1_ and for two cases: a) permeating monovalent and bivalent cations (left panel); b) permeating monovalent and trivalent cations (right panel).

Let us now assume that we have an ideal cation exchange membrane and that there are two permeating cations with two different charge numbers: a) monovalent and bivalent cations (z_1_ = 1, z_2_ = 2), or b) monovalent and trivalent cations (z_1_ = 1, z_2_ = 3). Furthermore, we assume that we deal with ideal solutions. In Fig 1 we show how in such cases the Gibbs-Donnan factor for the monovalent cations depends on the ratio of equivalents of non-permeating ions on the two sides of the membrane $\gamma_{\text{np},\text{2}}/\gamma_{\text{np},\text{1}}=Z_{np,2}C_{np,2}/Z_{np,1}C_{np,1}$. Note that when the monovalent cations are accompanied by trivalent cations (case b), their Gibbs-Donnan factor is accordingly higher (for abscissa range < 1) or lower (for abscissa range > 1), compared to the solution with bivalent cations (case a). If the ionic equivalents of non-permeating ions are equal at both sides of the membrane, $\gamma_{\text{np},\text{2}}/\gamma_{\text{np},\text{1}}=Z_{np,2}C_{np,2}/Z_{np,1}C_{np,1}=1$, then the Gibbs-Donnan factor is equal to 1. It is worth noting that, theoretically, one could approach the limit $\gamma_{\text{np},\text{2}}/\gamma_{\text{np},\text{1}}=Z_{np,2}C_{np,2}/Z_{np,1}C_{np,1}\to 0$ by having Z_np,2_C_np,2_ → 0 or Z_np,1_C_np,1_ → ∞, but actually the concentration of non-permeating ions in compartment 2 cannot be equal to zero if one wants to have permeating ions also in this compartment, because of the neutrality of the solution.

**Fig 2:**
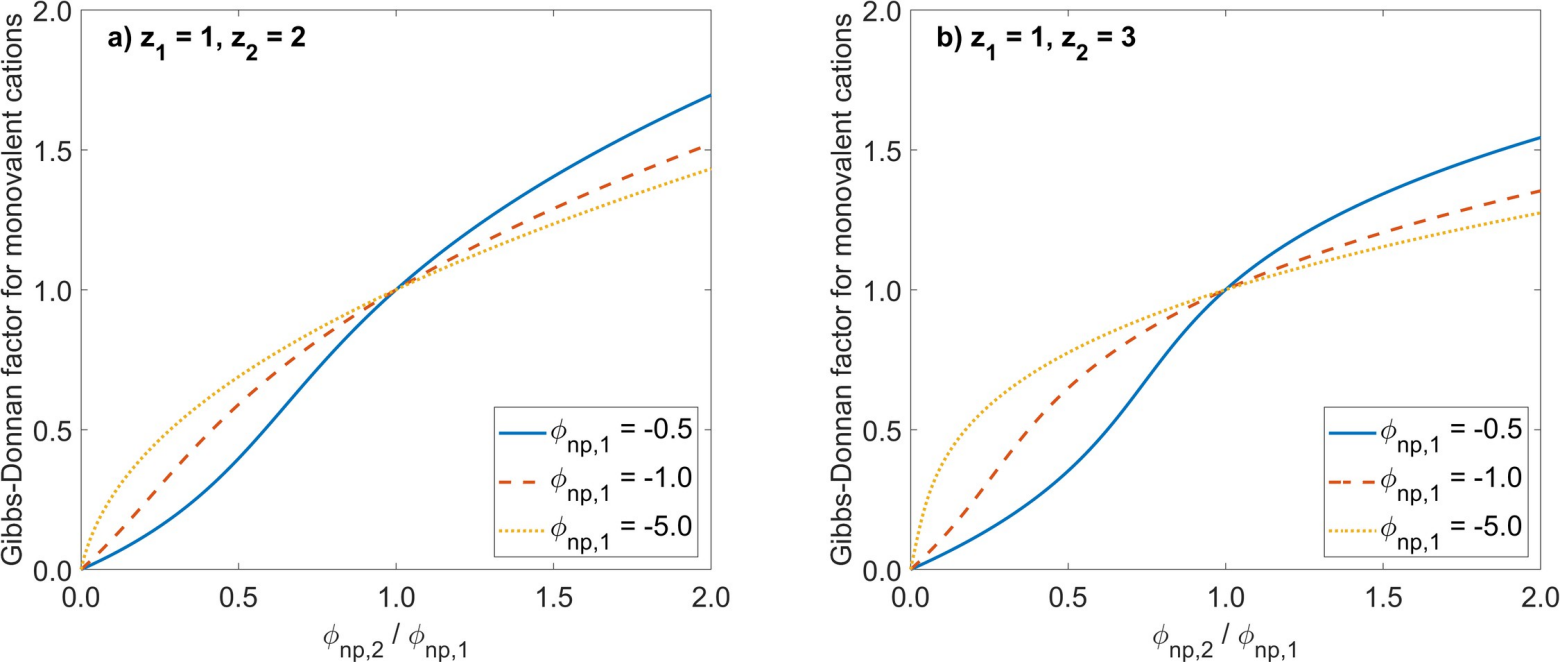
Gibbs-Donnan factors for different ratios of non-permeating ions on the two sides of a cation exchange membrane with known total masses of permeating ions. Shown are the relationships between the Gibbs-Donnan factor for a permeating monovalent cation and the ratio of equivalents of non-permeating ions on the two sides of a cation exchange membrane $\phi_{\text{np},\text{2}}/\phi_{\text{np},\text{1}}=Z_{np,2}C_{np,2}/Z_{np,1}C_{np,1}$, calculated according to Eq (45) (for the known total amounts of permeating ions distributed between the two compartments of the same volume, i.e., b = 1) for different values of ϕ_np,1_ and for two cases: a) permeating monovalent and bivalent cations (left panel); b) permeating monovalent and trivalent cations (right panel).

Fig 2 shows the analogous relationships as in Fig 1 but for the case when one knows the total amounts of permeating ions distributed between the two compartments (Eq (45)), and assuming the same volume of both compartments (b = 1). One can observe similar features of the presented curves as in the case presented in Fig 1; however for low absolute values of ϕ_np,1_ (e.g. ϕ_np,1_ = -0.5) the function has a deflection point, not observed for higher absolute values of ϕ_np,1_. The point with coordinates (1,1) is again the “meeting point” for all curves, as in Fig 1.

**Example 2.** *Permeating anions with the same charge numbers (a real case)*

The experimental data discussed in Examples 2 and 3 were collected during single runs for each set of initial concentrations and each membrane [8,13,21,26]. Therefore, the authors of those studies reported the results as single values and not as means and scattering around the mean (for example standard deviation). Our deterministic model also produces only one set of equilibrium values for each set of initial concentrations. Therefore, no statistical evaluation could be provided for these examples.

Let us now consider an anion exchange membrane with permeating anions with the same charge number, and let us assume that both compartments have the same volume (b = 1), which does not change during the ion exchange process (i.e. the equilibrium volumes are the same as initial volumes). For example, the transport of monovalent anions, such as phosphate, bicarbonate, and nitrate, across an anion exchange membrane was investigated in [8,26]. In one of those experiments, the initial receiver solution in compartment 1 was 17.1 mmol/L of NaCl, and the feed solution in compartment 2 was 5.03 mmol/L of KH_2_PO_4_ [8]. Thus, in our notation the initial data for the two permeating anions, i.e. Cl^-^ and H_2_PO_4_^-^, are as follows: charge numbers: z_1_ = -1, z_2_ = -1, initial concentrations (in mmol/L): c_1,1_ = 17.1, c_1,2_ = 0, c_2,1_ = 0, c_2,2_ = 5.03. The ionic equivalents of non-permeating ions, i.e. cations: Na^+^ and K^+^, are Z_np,1_C_np,1_ = 17.1 mEq/L and Z_np,2_C_np,2_ = 5.03 mEq/L. In this simple case of monovalent permeating ions, the Gibbs-Donnan factor can be calculated from Eq (18) as $DF_{2,21}=x=\frac{\gamma_{np,2}}{\gamma_{np,1}}=\frac{Z_{np,2}C_{np,2}}{Z_{np,1}C_{np,1}}=0.294$, and the equilibrium concentrations of phosphate can be calculated from Eq (37) as c_2,1_ = 3.89 mmol/L in the receiver compartment and then c_2,2_ = xc_2,1_ = 1.14 mmol/L in the feed compartment. Note that Eqs (35)-(37) describe a case with a known total mass of each permeating ion species (M_i_) and known volumes of both compartments at equilibrium (V_1_ and V_2_), but since in the present case both compartments have the same and constant volume, it is actually not necessary to know the volumes of the compartments or the total masses of the permeating ions but only to know their initial concentrations. In the actual experiment, as described in [8], the equilibrium was not attained and the experiment was finished at c_2,1_ = 3.22 mmol/L and c_2,2_ = 1.98 mmol/L. The experimental separation effectiveness, EF_exp_, was therefore 0.385, whereas the maximal theoretical separation effectiveness for an ideal IEM, EF_ideal_, would be 0.706; thus, the relative separation effectiveness, EF_exp_/EF_ideal_, was 0.544. The kinetic effectiveness factor, kEF, obtained in this experiment was 0.606, whereas the maximal (equilibrium) kEF_eq_, calculated for b = 1 (as in the experimental setup), was 0.773, see Section 2.2.

**Example 3.** *Permeating cations with two different charge numbers (real cases)*

Let us now consider a cation exchange membrane with two permeating cations (n = 2), a frequent case in membrane research. For instance, several Donnan dialysis experiments with mixtures of monovalent and bivalent cations were described in [13]. In Table 2 we first present two cases from the aforementioned study (Case I and Case II), for which, based on the initial concentrations of permeating ions and the equivalents of non-permeating ions in the two compartments, and given the same volume of both compartments (i.e. b = 1), we calculated the Gibbs-Donnan factors using Eq (45) and then the (ideal) equilibrium concentrations of permeating ions using Eqs (13) and (38).

**Table 2:** Examples of predicted Gibbs-Donnan equilibria for different ion solutions separated by a cation exchange membrane vs experimental results. Shown are exemplary cases of the predicted (calculated) Gibbs-Donnan equilibrium for ideal cation exchange membranes separating different mixtures with monovalent and multivalent cations (n = 2), acting as driving (d) and feed (f) ions, calculated using Eq (45) vs final concentrations of feed ions in the feed compartment obtained in the experimental setup.

			Case I [13]	Case II [13]	Case III [21]	Case IV [21]
initial data	charge numbers	z_1_	1 ^f^	1 ^d^	1 ^d^	1 ^d^
		z_2_	2 ^d^	2 ^f^	2 ^f^	3 ^f^
	initial concentrations of permeating ions, mol/L	c_1,1_	0.01	1.00	1.00	1.00
		c_1,2_	0.01	0.00	0.00	0.00
		c_2,1_	1.00	0.01	0.00	0.00
		c_2,2_	0.00	0.01	0.01	0.01
	equivalents of permeating ions, Eq/L	z_1_h_1_	0.02	1.00	1.00	1.00
		z_2_h_2_	2.00	0.04	0.02	0.03
	equivalents of non-permeating ions, Eq/L	Z_np,1_C_np,1_	-2.01	-1.02	-1.00	-1.00
		Z_np,2_C_np,2_	-0.01	-0.02	-0.02	-0.03
	relative ionic equivalents	ϕ_2_	100	0.04	0.02	0.03
		ϕ_np,1_	-100.5	-1.02	-1.00	-1.00
		ϕ_np,2_	-0.5	-0.02	-0.02	-0.03
calculated values	Gibbs-Donnan factors	DF_1,21_ = x	0.06631	0.02039	0.02040	0.03093
		$DF_{2,21}=x^{z_{2}/z_{1}}$	0.00440	0.00042	0.00042	0.00003
	ideal equilibrium concentrations of permeating ions, mol/L	c_1,1_	0.019	0.980	0.980	0.970
		c_1,2_	0.001	0.020	0.020	0.030
		c_2,1_	0.996	0.0199	0.0099	0.0099
		c_2,2_	0.004	8.3 x 10^−6^	4.2 x 10^−6^	3.0 x 10^−7^
experimental data	final concentrations of feed ions in the feed compartment, mol/L	c_1,2_	0.0025–0.0060*	N/A	N/A	N/A
		c_2,2_	N/A	0.0004	0.0008	0.0056

* depending on the combination of driving and feed ion species in the experiment (due to varied diffusion of various ions within the studied membrane).

In Case I, bivalent cations (Ca^2+^, Cu^2+^, or Mg^2+^), present initially only in compartment 1, were used as driving ions (i.e. ions whose transport across the membrane according to their concentration gradient “drives” the transport of feed ions in the opposite direction), and monovalent cations (H^+^, Na^+^, or K^+^) were the feed ions present initially both in the feed compartment 2 and at equal concentration in compartment 1 (see Table 2) [13]. In Case II, monovalent cations (Na^+^ or K^+^) were the driving ions present initially in compartment 1, and bivalent feed ions (Ca^2+^, Cu^2+^, or Mg^2+^) were present both in the feed compartment 2 and in compartment 1 at the same concentration (Table 2). The results of these experiments were presented graphically in the form of the ratio of the concentration of the feed ions in the feed compartment to their initial concentration in the feed compartment as a function of the duration of the experiment [13]. In Case I, the equilibration rate was rather low, and at the end of each experiment of around 5 h the concentration of monovalent feed ions in the feed compartment 2 was still decreasing (at different rates for different combinations of driving and feed ion species), thus remaining above the predicted ideal equilibrium concentration, see Table 2, [13]. In Case II, all combinations of driving and feed ion species were close to equilibrium already after 100 min of the experiment, and the measured concentration of the bivalent feed ions in the feed compartment 2 was above the predicted ideal equilibrium concentration, see Table 2, although the difference was not important from the practical point of view. Note that when driving ions are monovalent and feed ions are bivalent (Case II, as opposed to bivalent driving ions and monovalent feed ions, Case I), the feed ions are exchaned through the membrane both faster (as shown by the experiments) and more effectively (as indicated by the predicted ideal equilibrium concentrations).

Finally, in the last two columns in Table 2, we present two cases with bivalent and trivalent cations (Ni^2+^ as NiCl_2_ or Fe^3+^ as FeCl_3_) separated by a cation exchange membrane from HCl, as studied in [21]. Again, we used Eqs (13), (38) and (45) to calculate the Gibbs-Donnan factors and ideal equilibrium concentrations for both of those cases, i.e. Ni^2+^ and H^+^ (Case III) and Fe^3+^ and H^+^ (Case IV). Note that for the same driving ions (H^+^) and the same initial concentrations, the calculated ideal equilibrium concentration of trivalent cations (Fe^3+^) in the feed compartment 2 is lower than that of bivalent cations (Ni^2+^); however, in the 2-hour experiments the final concentration of Fe^3+^ was actually higher than that of Ni^2+^ [21], which suggests that the transport of Fe^3+^ across the studied membrane was considerably slower compared to Ni^2+^.

For the cases presented in Table 2, the maximal (equilibrium) kEF_eq_ for an ideal IEM (as defined in Section 2.2) were: 0.9091 for Case I, 0.9992 for Case II, 0.9996 for Case III, and above 0.9999 for Case IV, whereas the actual kEF values achieved at the end of each experiment (before the equilibrium was reached) were: 0.40–0.75, 0.96, 0.92, and 0.44, respectively (c.f. experimental data in Table 2). Thus, the experimental effectiveness was close to the ideal effectiveness in Cases II and III.

## 6. Discussion

The charge-based selectivity of IEMs (i.e. the discrimination between permeating and non-permeating ions according to the sign of their charge) leads to the exchange of permeating ions across the membrane towards an equilibrium with a non-uniform distribution of these ions on the two sides of the membrane that depends on the equivalents of non-permeating ions. This kinetic process can be mathematically modelled using differential equations [5,8,11,13,18,26–29]; however, one can also estimate the equilibrium concentrations of permeating ions using relatively simple algebraic equations, as shown in the present study. The assumption of ideal non-permeability of IEM to co-ions is of course only an approximation of reality, and hence the validity of the presented equations depends on the quality of the membrane and in particular on the difference between the rate of transport of permeating ions and the typically slow leakage of co-ions through the membrane.

The consideration of idealized physical systems is often applied in thermodynamics to elucidate the mechanisms of thermodynamic equilibria or processes and to obtain approximate estimates of certain thermodynamic parameters. Perhaps the best known example is the concept of a maximal efficiency of heat engines that can be obtained only in certain ideal conditions, as for example for the reversible Carnot heat engine [30,31]. Here we propose a method for evaluating the separation effectiveness of an idealized IEM that is fully impermeable to ions with the given charge sign (i.e. an ideal cation or anion exchange membrane). The real membranes can get close to this ideal case, but even very slow permeation of co-ions across the membrane would eventually lead to the equilibrium of all ions with equal concentrations on both sides of the membrane (assuming no adsorption or trapping within the membrane). The idealized IEM can serve as a reference membrane that allows for the estimation of how close the considered real membrane is to the ideal one for the investigated ionic system. Note that, so far, there seems to be no proof that the proposed description of the ideal separation effectiveness of an IEM calculated from the Gibbs–Donnan factor provides the maximal possible separation effectiveness, although the experimental data discussed in the Examples section may suggest such a conjecture.

The problem of equilibrium in an IEM system was discussed in two versions. The first one, related to the classic Gibbs–Donnan equilibrium, was aimed at the estimation of the Gibbs–Donnan factors with the assumption that one knows all ion equilibrium concentrations on one side of the IEM and only the concentrations (or equivalents) of non-permeating ions (co-ions) on the other side of the membrane. Note that the non-permeating ions are always present on both sides of the membrane because of the electric charge neutrality condition. This problem was reduced to solving a single algebraic Eq (10). In general, solving this equation is not trivial, although closed mathematical solutions can be obtained for some specific ideal cases, as for example if all permeating ions have the same charge number, see Eq (18). If there are permeating ions with two different charge numbers, one already needs numerical solutions, as shown in Fig 1. In general, Eq (10) considers non-ideal solutions with ion activities and their activity coefficients. As an example of the calculations for non-ideal systems, we presented the equations based on the Debye–Hückel theory, and we provided examples of such calculations in Table 1.

The second version of our approach addresses the problem of estimating the equilibrium distribution of permeating ions across an IEM if their total mass in the system is known (together with the concentrations or equivalents of non-permeating ions), which also uses the general formula for the Gibbs–Donnan factor, Eq (12). In such a case, again, closed formulas may be obtained for some simple ideal solutions, but in the general case, a numerical solution is needed, as shown, for example, in Fig 2. In Table 2, we compared the numerical predictions of Gibbs-Donnan equilibria to some experimental data and discussed the impact of various combinations of feed and driving ions on the separation effectiveness of an IEM.

Some additional assumptions are used in our theoretical considerations. In particular, we assume that there is no chemical gradient caused by a hydrostatic or hydraulic pressure difference across the membrane (here, we consider only the electro-diffusive transport of ions and not the possible osmotic shifts of the solvent across the membrane) nor any external electrostatic gradient across the membrane. For a more general approach to the Gibbs–Donnan equilibrium that includes osmotic pressure, see [32].

We also assume that all considered permeating ions are present only in fully dissociated form, i.e., that they do not form any chemical compounds (pairs or complexes) with their counter-ions. For the remarks on the case with ions present in various chemical forms, see our earlier work [19]. We also assume that none of the permeating ions are bound to non-permeating ions; in other words, we assume that c_i_ denotes the concentration of the free-fraction of ion i in the mixture. Moreover, we assume that the permeating ions do not adsorb on the membrane nor are trapped within the membrane. All these assumptions can be replaced by additional equations in the theory, if more detailed information on the system is available, although at the cost of more sophisticated mathematics and calculations. A more advanced approach would be needed for the description of membranes that combine size- and charge selectivity, such as nanofiltration membranes, see for example [33].

Another approach to calculating (estimating) the equilibrium concentrations of ions at the two sides of an ideal IEM would be mathematical modeling of transient states until the solution is numerically close to the equilibrium. We are, however, not aware of such studies. The result of such an approach may depend on the assumptions used in the employed kinetic model. In contrast, our approach is based only on the general rules of charge neutrality and mass balance and basic thermodynamic equilibrium formulae. It may be formulated for ideal solutions but also for more realistic cases of non-ideal solutions, using an appropriate theory for such solutions, as we have shown here using the Debye-Hückel theory.

## 7. Conclusion

We conclude that the Gibbs–Donnan equilibrium approach is theoretically and numerically feasible for the assessment of systems with ion exchange membranes and may be helpful for predicting the boundary values for the equilibrium concentrations of driving and feed ions and for assessing the effectiveness of ion separation in various processes involving IEMs.
